# Genes Relevant to Tissue Response to Cancer Therapy Display Diurnal Variation in mRNA Expression in Human Oral Mucosa

**DOI:** 10.5334/jcr.213

**Published:** 2021-06-17

**Authors:** Fangyi Gu, Eduardo Cortes Gomez, Jianhong Chen, Matthew F. Buas, Nicolas F. Schlecht, Karen Hulme, Shweta Vishwas Kulkarni, Prashant K. Singh, Richard O’Connor, Christine B. Ambrosone, Anurag K. Singh, Jianmin Wang

**Affiliations:** 1 Department of Cancer Prevention and Control, Roswell Park Comprehensive Cancer Center, US; 2 Department of Biostatistics and Bioinformatics, Roswell Park Comprehensive Cancer Center, US; 3 School of Public Health, University of Buffalo, US; 4 Center for Personalized Medicine, Roswell Park Comprehensive Cancer Center, US; 5 Department of Radiation Medicine, Roswell Park Comprehensive Cancer Center, US

**Keywords:** circadian rhythm, chronotherapy, biomarkers, oral mucosa, cancer therapy

## Abstract

**Background:**

To address a critical gap for application of cancer chronotherapy of when would be the best time(s) for treating an individual cancer patient, we conducted a pilot study to characterize diurnal variations of gene expression in oral mucosal tissue, which is vulnerable to damage from cancer therapies.

**Methods:**

We conducted RNA-seq assay on individual oral mucosal samples collected from 11 healthy volunteers every 4 hours (6 time points). Using a cosine-based method, we estimated the individual and average values of peak-time and amplitude for each gene. Correlations between gene expression peak-times and age was examined, adjusting for individual’s sleep timing.

**Results:**

Among candidate gene pathways that are relevant to treatment response, 7 of 16 genes (*PER3, CIART, TEF, PER1, PER2, CRY2, ARNTL*) involved in circadian regulation and 1 of 118 genes (*WEE1*) involved in cell cycle regulation achieved p-value ≤ 0.1 and relative amplitude>0.1. The average peak times were approximately 10:15 for PER3, CIART and TEF, 10:45 for PER1, 13:00 for WEE1, PER2 and CRY2, and 19:30 for ARNTL. Ranges in peak times across individuals differed by gene (e.g., 8 hours for PER1; 16.7 hours for WEE1). Older people had later peak times for PER1 (r = 0.77, p = 0.03) and PER3 (r = 0.69, p-value = 0.06).

**Conclusion:**

In oral mucosa, expression of some genes relevant to treatment response displayed diurnal variation. These genes may be candidates for development of biomarkers for optimizing individual timing of cancer therapy using non-invasively collected oral mucosa.

## Background

The importance of circadian regulation in both chemotherapy [1] and radiation therapy is increasingly well documented [2]. Conducting cancer therapy at optimal times of day based on circadian variations of sensitivity to cancer treatment (chronotherapy) has great potential for improving therapeutic efficacy and/or decreasing side effects [1,2]. Oral mucositis (OM) is a common debilitating complication of cancer chemo- and radiotherapy occurring in nearly all patients with head-and-neck cancer and causes significant morbidity and reduced therapeutic effectiveness [3,4]. With limited prevention and treatment options, there is an urgent need to develop novel strategies for preventing or mitigating OM to improve patients’ outcomes and quality of life [3]. Treatment-induced OM is typically seen as an “outside-in” process, in which chemo- or radiotherapy nonspecifically targets the rapidly proliferating cells of the basal epithelium, causing the loss of ability to self-renew, inflammation and ulceration [5]. We and others have shown that the severity of OM may depend on time of radiotherapy [2,6,7]: patients treated in the morning have less severe OM. These findings are biologically plausible, as many key regulators of fundamental biological processes that influence tissue response to cancer therapy, such as cell cycle progression [8,9,10,11] and DNA damage response [12], are under circadian control [13]. Therefore, choosing treatment times associated with less radio-sensitivity in non-cancerous oral mucosa could be one option for reducing severity of radiation-induced OM.

The application of chronotherapy is complicated by inter-individual variability of circadian phase in humans and differences between central (i.e., in the suprachiasmatic nucleus) and peripheral (eg., oral mucosal tissue) circadian phase. The Dim Light Melatonin Onset (DLMO) is currently considered to be the most accurate marker of the circadian phase in humans [14]. However, there are two problems with using it as a marker of circadian phase for chronotherapy: (i) determining DLMO requires multiple consecutive samples of blood or saliva collected across an 8- to 24-hour interval under controlled conditions and (ii) DLMO represents the central clock time (“phase”) [14], which differs from the circadian clock time in peripheral tissues [15] that are vulnerable to the damage of cancer therapy (both chemotherapy and radiation).

Critical determinant of sensitivity of normal cells to radiation therapy is the cell cycle phase: cells in the G1 and S phases are less radio-sensitive than those in the G2/M phases [16,17]. Circadian rhythms in mitotic index and DNA synthesis, the major events in the M and S phase of a cell cycle, have been documented in epithelium of mouse alimentary tract (intestine, tongue, esophagus and stomach) [18,19], as well as in human rectal and oral mucosa [20,21]; these findings provide a foundation for using markers of cell cycle progression as potential biomarkers for development of personalized chronotherapeutic schedules for cancer treatment. Previous study analyzing repeated oral mucosal samples from six healthy male volunteers has reported diurnal variation of five cell-cycle-associated proteins (Cyclin E, A, B1, Ki-67 and p53) [22]. However, the sample collection method (invasive punch biopsy) limited the potential for future clinical application.

In this pilot study, we assessed diurnal variation in mRNA expression of genes relevant to treatment response using oral mucosa samples collected by a non-invasive brush biopsy, in healthy volunteers [23]. Factors that may impact inter-individual difference of circadian timing were also examined. This study was designed for examining diurnal rhythm (i.e., endogenous circadian plus potential driven effects from behavior cycles such as the sleep-wake and feeding cycle), rather than endogenous circadian rhythm (purely driven by endogenous pacemaker); the latter requires a burdensome constant routine protocol to eliminate all periodic changes in behavior and environment [24]. By allowing the participants to follow their usual schedule of sleep and meal will generate the data that reflex participants’ real-life experience, and therefore has better potential for translation to clinical setting.

This work is a fundamental first step in developing biomarkers for determining an optimal timing of cancer treatment for an individual patient to reduce oral mucositis by establishing whether the expression of these genes is rhythmic across the 24-h day.

## Methods

### Study population

We recruited 11 healthy volunteers, 3 men aged 23–50 and 8 women aged 24–60, with no history of cancer diagnosis, through the University at Buffalo (UB) Clinical and Translational Science Institute (CTRC) Community Recruitment Liaison, and an email circulated to UB students. Exclusion criteria for the study included: pregnant females or females using oral contraceptives or hormonal therapy; presence of open blisters, sores or lesions in the mouth that would make brushing of the buccal mucosa difficult or painful; current self-reported sleep disorders (e.g., sleep apnea, delayed sleep phase disorder, advanced sleep phase disorder); psychiatric conditions (i.e., depression or anxiety); color blindness (as determined by the Ishihara test) [25]; current smoking (e.g., every day or some days); drug use (e.g., marijuana, opioids, cocaine, amphetamine); inability to stop drinking coffee or alcohol for 24 hours before and during sample collection; use of any medications that may influence sleep or circadian rhythm within three weeks before and during the sample collection; working the night shift within at least two months prior to the study; and traveling across more than one time zone in the month preceding the study. Participants provided written informed consent prior to their participation. The study was approved by the Roswell Park Comprehensive Cancer Center Institutional Review Board. This study is not a clinical trial, therefore is not required to be registered on *Clinicaltrials.gov*.

### Study design and data collection

Each participant completed the Pittsburgh Sleep Quality Index questionnaire [26] and the Morning-Evening Questionnaire [27] at consent and was asked to wear a wrist actigraphy monitor (Actiwatch Spectrum; Philips Respironics, Oregon, USA) on the non-dominant arm. This actigraphy monitor, together with a sleep log, were used to monitor the participant’s sleep-wake cycle for one week prior to sample collection [28]. The time interval between actigraphy sleep data collection and laboratory sample collection ranged between 2 days to 32 days (<7 days for 5 participants, 11 days for one, 14 days for three, 21 days for one, and 32 days for one participant).

**Figure 1:**
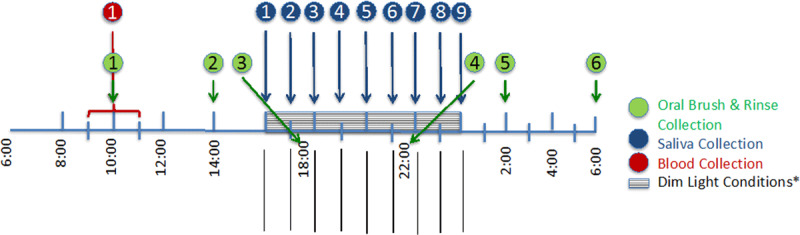
Sample collection schedule of healthy participants. Participants arrived at the CTRC 9:00–9:30 on scheduled date and stayed in the room until about 6:00 the next day for sample collection. From each participant, cheek cells were collected every four hours (green) between about 10:00 on day 1 and 6:00 on day 2; saliva was collected every hour, starting from about 6 hours before their usual bedtime (10 p.m. in this example) until 2 hours after their usual bedtime, under dim light (light exposure lower than 5 lux). Blood was collected about 10:00.

Volunteers were asked to arrive at the CTRC between 9:00 and 9:30 on the scheduled date. They stayed in a private CTRC room for sample collection for approximately 21 hours until about 7:00 next day; each private room had an examining table and chair, a reclining chair, a hospital bed for overnight stays, and windows with light filtering curtains. A detailed sample collection protocol is summarized in ***Figure 1***. Briefly, six samples of full layers of cheek buccal cells were collected by a trained staff member every 4 hours, from about 10:00 to 6:00 the next day using the OralCDx® brush (CDx Diagnostics, NY), a non-invasive method for collecting cells from all epithelial layers, typically used in dental offices for early detection of pre-cancer [23]. In parallel, saliva samples were collected every hour, starting from about 6 hours before the participant’s usual bedtime until 2 hours after their usual bedtime. Saliva was collected before cheek cell samples when collection times of saliva and cheek overlap each other. Throughout this time interval, samples were collected under dim light conditions (<5 lux). Saliva samples were collected using Salivette cotton (Sarstedt, Newton, NC) that participants were instructed to put into their mouths and chew for about 1 min until saturated, before spitting it back into the Salivette. No saliva sample was contaminated by blood, as determined by checking the color of saliva. Food was not allowed within 15 minutes before saliva sample collection. After each meal or snack, participants were instructed to brush their teeth using water without toothpaste. Tooth brushing, rinsing or water drinking was not allowed within 10 minutes before saliva collection. Volunteers were allowed to maintain their usual activities and take normal meals; they were encouraged to read, write, and listen to music while in the facility. During sleep time the rooms were dark; a dim light was allowed at the time of sample collection and when participants needed light in the room.

### RNA extraction and RNA-seq assay

Immediately following each brush mucosal cell collection, the brush was immersed in Qiazol (cell lysis Reagent from Qiagen, USA), mixed well, and stored temporarily in a –80° C freezer at CTRC-CRC. The next day, all the completed brush mucosal samples were delivered on dry ice to Roswell Park’s DataBank and BioRepository (DBBR) Shared Resource for storage at –80° C. After sample collection was completed from all participants, they were delivered on dry ice to the Roswell Park Genomics Shared Resource for RNA extraction and RNA-seq assay. Purified RNA was prepared using the miRNeasy micro kit (Qiagen, Cat No. 217084) and sequencing libraries were prepared with the KAPA RNA HyperPrep Kit with RiboErase (HMR) (Roche, USA), from 100ng total RNA. The final RNA-seq libraries were sequenced on Illumina NovaSeq 6000 using 100 paired end sequencing.

### Quality Control and bioinformatics analysis of RNA-seq data

Bioinformatics pre-processing and quality control (QC) steps were carried out by the Roswell Park Bioinformatics Shared Resources, using the established pipeline following commonly adopted practices for RNA-seq data analysis. Raw reads that passed the Illumina RTA quality filter were demultiplexed and pre-processed by using FastQC for sequencing base quality control. The reads were then mapped to the latest version of human reference genome (GRCh38) and reference transcriptome GENCODE (v25) using Bowtie (v1.0.1) [29] and TopHat (v2.0.13) [30] aligner. A second round of quality control was performed to identify problematic samples with RNA-seq library preparation using the RSeQC (v2.3.2) [31]. Because oral mucosal samples contain bacteria, which will decrease the mRNA integrity number (mRIN), we additionally checked gene body coverage of housekeeping genes to better assess the integrity/degradation of the human RNA samples. Transcripts Per Kilobase Million (TPM) estimates for gene expression were obtained using Salmon (v0.13.1). A total of 19798 genes had detectable expression data and were qualified for further analysis. Log10 transformed TPM values with a 0.1 correction constant were used for harmonic gene testing (MetaCycle).

### RNA quality

The RNA qualities from all samples were acceptable with over 3 million reads obtained to quantify gene expression. The mRIN ranged from 2.0 to 6.8 (Supplementary Figure 1.), acceptable for whole transcriptome (ribosomal depletion) library preparation. The mapped reads were evenly distributed onto different gene features (CDS exons, UTRs) and the coverage across whole gene body did not show obvious significant 3’ or 5’ bias for any sample (Supplementary Figure 2a.). Due to the nature of sample collection site (i.e., oral cavity), microbiome RNA contamination was inevitable, and the mapping rates reflect the amount of foreign RNA in the sample (Supplementary Figure 2b.). To better assessing the RNA integrity, Supplementary Figure 3. showed that the house keeping gene body coverages were overall uniform in all samples, which means that there was no obvious degradation of human RNA happening in any sample. So, the low RIN (<4) for some samples is primarily caused by microbiome RNA and does not reflect the sample quality. After mapping and assigning reads to human genome reference using Salmon, a mean of 10,689,349 and sd of 8,075,814 reads were quantified in total.

### Melatonin assay and DLMO estimation

Saliva samples were centrifuged immediately to extract the saliva from the cotton swab and then frozen at –80° C temporarily at CTRC-CRC. The next day, all saliva samples were delivered on dry ice to Roswell-DBBR for storage at –80° C. After completing field sample collection from all participants, saliva samples were shipped on dry ice to Solidphase, Inc. (Portland, ME) for melatonin radioimmunoassay using commercially available kits (BUHLMANN, Schönenbuch, Switzerland). Based on duplicate quality control samples, the intra-assay and inter-assay CVs were 1.9% and 6.6%, respectively, for the low concentration samples (range 1.6–4.3 pg/ml); 8.0% and 0.9%, respectively, for the high concentration samples (range 16.7–31.1 pg/ml). Circadian phase was defined as the clock time at which melatonin concentration exceeded the threshold of 3 pg/mL, indicating the onset of melatonin secretion under dim light conditions [Dim Light Melatonin Onset (DLMO)] estimated by linear interpolation based on the clock time of two consecutive samples with melatonin concentration less than and greater than the threshold, respectively. Specifically, if the two known values for the adjacent times around the threshold were (t_1_, m_1_) and (t_2_, m_2_), then the time of {\rm DLMO} = {t_1} + ({\rm threshold}-{m_1}) {\textstyle{{{t_2}-{t_1}} \over {{m_2}-{m_1}}}}.

### Statistical analyses

To identify genes whose expression had a significant variation by time of a day, we used a cosine model implemented by the MetaCycle R package [32]. The MetaCycle estimates circadian parameters from three independent rhythm detecting models (ARSER, JTK_CYCLE and Lomb-Scargle) and integrates them using a voting scheme. The three methods considered by MetaCycle have their own strengths and weaknesses depending on how the data was generated. In the case of the present study, the data are continuous, unevenly spaced with 6 time points per subject which better suits the features covered by the Lomb-Scargle method. Briefly, Lomb (1976), proposed a model using least-squares to fit sinusoidal curves. Scargle (1982) extended this method by defining a Lomb-Scargle periodogram and obtaining the null distribution for it [33]. This fit is conducted on the gene expression data of six time points by each gene and participant. Resulted estimates of circadian parameters for each participant were then integrated using the MetaCycle’s meta3d function.

Meta3d can directly output the p-value of rhythmic signal, phase, amplitude, relative amplitude values (rAMP) and baseline value. Amplitude is half the difference between the estimated minimum and maximum values (23 and 25 were set for this study) Relative amplitude values are the ratio of the amplitude and baseline value, which may be used to compare the amplitude values among genes with strongly different expression levels. Group-level average phase was calculated using the circular mean from each participant’s phase estimate. For details of parameter estimation in Meta3d, refer to the link (*https://cran.rproject.org/web/packages/MetaCycle/vignettes/implementation.html*). We selected *a priori* candidate genes involved in circadian regulation, cell cycle progression (a strong predictor of radio-sensitivity), and DNA homologous recombination repair (a critical pathway for the repair of DNA double-strand breaks). We focus on these pathways because they are relevant to circadian regulation and/or the response to cancer therapy [16,17]. The gene list included 16 well-established circadian genes with noted circadian variation in human skin and mouse tissues [34]: ARNTL, NPAS2, CLOCK, PER1–3, CRY1–2, CIART, NR1D1, BHLHE41, DBP, TEF, HLF, RORC, NFIL3. The genes for the cell cycle progression (n = 118) and homologous recombination DNA double-strand break repair (n = 26) were based the Molecular Signature Database [35] using c2-CP (curated gene set – canonical pathways). According to a methodological guideline for genome-scale analysis of biological rhythms [36], we used different thresholds to define whether a gene mRNA expression was rhythmic. We present major findings based on the criteria of p-value ≤ 0.1 and relative amplitude (rAMP) > 0.1. False discovery rate [37] was then used to adjust for multiple comparisons for number of genes examined in each pathway.

To evaluate the peak time variability of these diurnally expressed genes, we calculated the range of point estimate for gene peak times, relative to the wall clock time, circadian phase and sleep time. The gene expression peak times relative to circadian phase or sleep time were calculated by subtracting the circadian (DLMO) time or the midpoint of sleep-onset and wake-up (midsleep) time from original peak times.

We further assessed the circular correlation between the peak times of identified genes and phases of other biological rhythms (the DLMO time and midsleep time) using the CircStats R package [38]. Specifically, the “circ.cor” function was employed to estimate a circular version of Pearson’s product moment correlation. Distribution of variables were examined using goodness of fit test from the package to ensure the applicability of the correlation calculation.

To examine correlation between gene peak times and characteristics of participants (age, sleep time), we used Partial Spearman correlation tests (SAS 9.4). Each analysis adjusted for covariates of age, sex, sleep time, and DLMO, excluding the variable that was being examined.

Using gene expression data of all 66 samples from 11 participants, we examined the correlation between mRNA expression of circadian genes and *WEE1*, a gene involved in G2/M cell cycle checkpoint inhibition, using repeated measures correlation method accounting for intra-participant variation as implemented in R package rmcorr (v0.3.0) [39]. Correlation values are displayed and organized using complete Euclidean clustering, and the heatmap was plotted using heatmap R package.

## Results

### Participants’ characteristics

Demographic information about the study participants, including age, sex, race, chronotype and sleep times is summarized in ***Table 1***. We studied 8 women and 3 men, 4 different ethnicities, ages ranging from 23 to 60 years. Based on the Morningness-Eveningness questionnaire [27], 9 participants belong to moderate morning or intermediate chronotype, 1 to moderate evening and 1 to definite morning type. The DLMO ranged from ~17:30 to 22:00. The PSQI-based bedtimes were between 20:30 and 1:30, wake-up times were between 5:45 and 7:45, and midsleep times (between bedtime and wake-up time) were between 1:15 and 3:15. Actigraphy-based wake-up time ranged from 5:31 to 7:57 on weekdays and from 3:19 to 8:40 on weekends. The midsleep time ranged from 1:56 to 4:07 on weekdays and from 23:28 to 6:01 on weekends.

**Table 1:** Table 1 Demographic information of study participants.


VARIABLES	NO.	%	MEAN (STD), MIN, MAX

Age (years)			

23–30	4	36.4	

31–50	4	36.4	

51–60	3	27.3	

Sex			

Women	8	72.7	

Men	3	27.3	

Race			

Asian	2	18.2	

Black	3	27.3	

Hispanic	1	9.1	

White	5	45.5	

Chronotype defined by the morning-evening questionnaire scores (Horne and Ostberg, 1976, ref 23)			

Moderate evening: 31–41	1	9.1	

Intermediate: 42–58	4	36.4	

Moderate morning: 59–69	5	45.5	

Definite morning: 70–86	1	9.1	

DLMO (3pg/ml) time (HH;MM)			20:13 (1:25), 17:22, 22:04

Bedtime-PSQI^*^ (HH:MM)			22:42 (1:18), 20:30, 25:30

Waketime-PSQI^*^ (HH:MM)			6:24 (0:36), 5:45, 7:45

Midsleep-PSQI^*^ (HH:MM)			2:24 (0:42), 1:15, 3:15

5-day average weekday midsleep – Actigraphy^**^ (HH:MM)			3:01 (0:48), 1:56, 4:07

2-day average weekend midsleep – Actigraphy^**^ (HH:MM)			3:39 (1:46), –0:28, 6:01

5-day average weekday waketime – Actigraphy^**^ (HH:MM)			6:25 (0:47), 5:31, 7:57

2-day average weekend waketime-Actigraphy^**^ (HH:MM)			7:16 (1:17), 3:19, 8:40

Intervals between completion of actigraphy data collection and sample collection			

<7 days	5	45.5	

11	1	9.1	

15–19	3	27.3	

>21	2	18.2	


DLMO: Dim Light Melatonin Onset based on thresholds of 3pg/ml. ^*^ Sleep times from the Pittsburg Sleep Quality Index Questionnaire responses. ^**^ Sleep times based on the actigraphy measurements over seven days.

### Genes with diurnal variation based on cosine model

The number of genes that can be defined as displaying diurnal variation depended on significance thresholds. Out of 19798 genes detected, 3 genes had p-value < 0.01, 108 genes had p-value < 0.05, 650 genes had p-value < 0.1, 2800 genes had p-value < 0.2 and 5514 genes had p-value < 0.3. Among the candidate pathway genes selected *a priori*, the three genes with the lowest p-values, *PER3, CIART*, and *WEE1* reached a criterion of p-value < 0.01 and relative amplitude (rAMP) > 0.1 (***Table 2***). After multiple comparison adjustment for number of genes tested in each pathway, *PER3* and *CIART* remained significant (FDR < 0.1). In addition, the circadian pathway genes *PER1, ARNTL, TEF, CRY2*, and *PER2* had p-value ≤ 0.1 and rAMP > 0.1 (***Table 2***). The average peak times of RNA expression were approximately 10:15 for *PER3, CIART* and *TEF*, 10:45 for *PER1*, followed by *PER2, CRY2* and *WEE1* peaking about 13:00; whereas *ARNTL* peaked about 19:30. Results of all candidate genes in the three pathways and the 108 genes with p-value < 0.05 are presented in Supplementary Table 1.

**Table 2:** Genes in the circadian and cell cycle pathways with p-value ≤ 0.1 and amplitude > 0.1.


GENE	AMP	RAMP	CLOCK PEAK TIME*				PEAK TIME RANGE			P-VALUE
	
			POINT ESTIMATE	AVERAGE	95%CI		TIME IN CLOCK	DLMO-ADJ**	MIDSLEEP-ADJ**	

*CIART*	0.36	0.66	10.4	10.3	8.7–11.8		6.7–14.5 (7.8)	10.7	9.3	0.008^†^

*WEE1*	0.18	0.21	12.5	12.9	9.6–16.2		2.1–18.8 (16.7)	15.1	15.6	0.009

*PER3*	0.27	0.50	10.0	10.1	8.6–11.6		6.3–14.4 (8.1)	8.3	8.6	0.009^†^

*PER1*	0.3	0.22	10.8	10.6	8.8–12.4		6.2–14.2 (8.0)	10.3	9.2	0.08

*ARNTL*	0.34	1.3	19.5	19.4	15.8–22.6		13.8–30.0 (16.2)	18.7	17.8	0.08

*TEF*	0.44	–1.76	10.3	11.4	9.0–13.7		6.7–18.1 (11.4)			0.09

*CRY2*	0.25	0.32	13.5	13.0	11.0–14.9		8.0–17.5 (8.5)			0.09

*PER2*	0.31	0.51	13.1	12.5	10.5–14.6		6.5–18.0 (11.5)			0.1


AMP: the amplitude is the half difference between the highest and lowest values of the log10(TPM) gene expression levels, based on the fitted MetaCycle harmonic model. rAMP (the relative amplitude): the ratio between the amplitude and baseline values (when the baseline absolute value is greater than 1). * Peak time point-estimate was based on the aggregated peak-time calculated using meta3d output; Average and 95%CI was based on individual participant peak-time values. ** Peak times of gene expression adjusted by DLMO or Midsleep for each participant was calculated by the gene peak time in clock minus each person’s DLMO time or PSQI-midsleep time. ^†^ False Discovery Rate < 0.1 adjusting for number genes in the circadian rhythm pathway.

**Figure 2:**
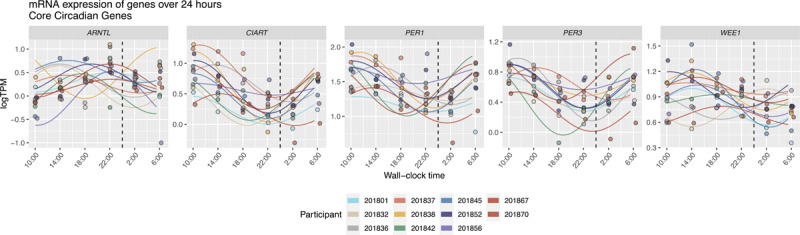
mRNA expression levels (LogTPM) over 20 hours for selected genes with most significant circadian variation. Numbers are Participant ID.

**Figure 3:**
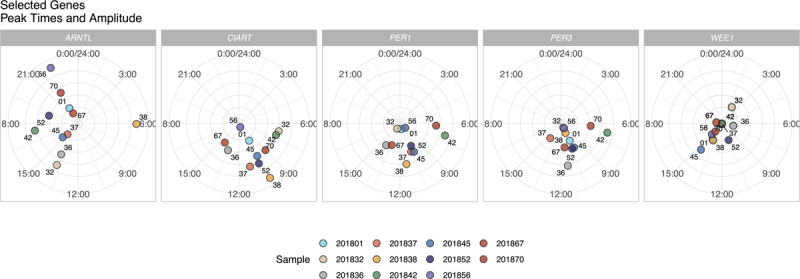
Estimated peak times and amplitudes of selected genes for each participant (n = 11). Peak times are indicated by the clock times; amplitudes are indicated by the distance from the center. The numbers are the last two digits of the Participant IDs listed in Figure 2.

The mRNA gene expression patterns over 20 hours of data collection (i.e., 10AM – 6AM) for the most significant five genes (*CIART, WEE1, PER3, PER1*, and *ARNTL)* are presented in ***Figure 2***. The peak times and amplitudes varied across participants (***Figure 3***). The range of peak time was 7.8 hours for *CIART*, 16.7 hours for *WEE1*, 8.1 hours for *PER3*, 8.0 hours for *PER1*, and 16.2 hours for *ARNTL* (***Table 2***) The range of the relative gene peak times adjusted for the DLMO and midsleep times was larger for *CIART, PER3, PER1 and ARNTL* and smaller for *WEE1* (***Table 2***). The range of peak times are greatly impacted by a few outliers: removing one participant who had outlying peak times reduced the average peak times for the study sample to 8.5 hours for *ARNTL* (***Figure 3***). For *PER1*, eight participants had peak times between 10:30 and 14:10 and three participants had peak times at 6:30, 7:00 and 9:00.

### Correlations of mRNA expression levels among genes in the circadian pathway

**Figure 4:**
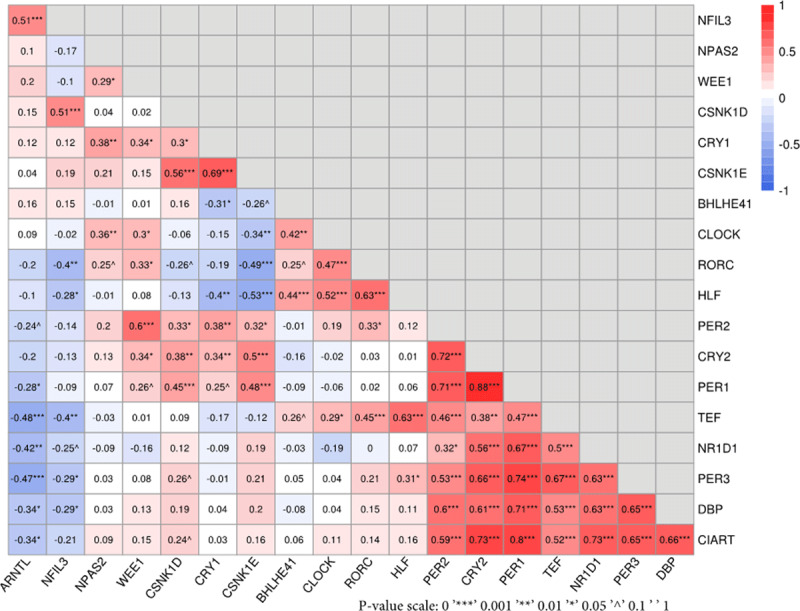
Heatmap of mRNA gene expression among genes in the circadian rhythm pathway and *WEE1*. Correlation coefficients are indicated using both numbers and colors.

The mRNA expression levels for *PER1, PER2, PER3, CRY2, CIART, TEF, DBP* and *NR1D1* were positively inter-correlated with each other (p-value < 0.05) and clustered together (Heatmap in ***Figure 4***). *ARNTL* was negatively correlated (r = –0.48 – –0.20) with the above-mentioned genes. *WEE1* was positively correlated with *PER2* (r = 0.6, p-value < 0.001), but had weaker correlation (r = 0.29–0.34, p-value < 0.05) or no correlation (p-value > 0.05) with other genes in the circadian pathway (***Figure 4***).

### Correlations between gene peak times, DLMO and sleep times, and age

**Figure 5:**
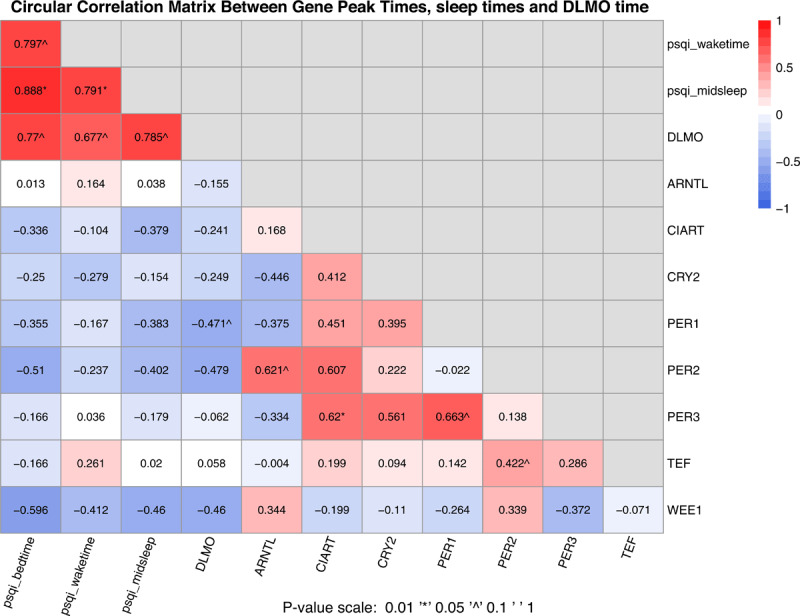
Correlations between peak times of selected circadian genes, with sleep times and the DLMO time.

The correlations between peak times of genes with diurnal variation (***Table 2***), sleep time and DLMO time are shown in ***Figure 5***. DLMO time was significantly correlated with sleep times collected by questionnaire (r = 0.68~0.79). There were also positive correlations for peak times of gene expression between *CIART* and *PER2, CIART* and *PER3, PER1* and *PER3*, as well as *ARNTL* and *PER2* (r = 0.61~0.66). However, peak times of genes with diurnal variation were not correlated with DLMO or sleep times, except for a negative correlation between *PER1* and the DLMO time.

The peak times of PER1 and PER3 were strongly correlated with age; older people tended to have later peak times after adjusting for gender, DLMO and sleep time (Partial Spearman correlation = 0.77 and 0.69, respectively) (***Table 3***, Supplementary Figure 4). We also found that older age has a tendency to be associated with earlier sleep time (r = –0.38) and earlier DLMO time (r = –0.4), however the small number of samples analyzed did not allow reaching statistical significance (Supplementary Figure 4). No correlation was observed between gene peak times and sleep time (***Table 3***).

**Table 3:** Partial Spearman correlation between gene peak times and age, and sleep time*.


		*PER1*	*PER2*	*PER3*	*CIART*

**age**	Coefficient	**0.77**	–0.50	**0.69**	0.25

	p value	**0.03**	0.21	**0.06**	0.55

PSQI_midsleep**	Coefficient	0.23	–0.27	–0.43	–0.48

	p value	0.58	0.52	0.29	0.23


* Analyses adjusted for age, sex, sleep time, and DLMO, excluding the variable that was being examined. ** mid-time between the bedtime and wake-up time from the Pittsburg Sleep Quality Index Questionnaire.

## Discussion

Using oral mucosal samples collected over 20 hours in 11 healthy volunteers by a non-invasive brush biopsy, we found that several genes involved in circadian rhythm regulation and cell cycle progression display time-of-day variations in their pattern of expression. Out of these genes, *WEE1* and *PER1 are of most interest*, which play direct roles in cell cycle progression and apoptosis, and associate with cancer therapy response; the finding of *WEE1* rhythmicity, previously reported in liver tissue of mice [40] and gut epithelium of rats [40,41], has never been reported in human oral mucosa. Peak times of oscillating genes differed by individuals; such variability across individuals could at least partially be explained by age after adjusting for sex, sleep time and DLMO.

We found that the peak times for mRNA expression of *PERs* and *CRY2* ranged from 10:00–13:30 across individuals, and ARNTL peaked on average at 19:30; there were strong positive correlations in mRNA expression of the *PERs* and *CRY2* genes, and negative correlations with *ARNTL* mRNA expression. Similar peak time order and correlations of these circadian core clock genes were reported previously in human skin [34]. These findings are consistent with the molecular mechanism of circadian rhythms, which is generated by an autoregulatory transcriptional feedback loops through positive and negative elements [42]. In mammals, during the day, CLOCK (or NPAS2) interacts with BMAL1 to activate transcription of the *PER* and *CRY* genes, resulting in high levels of these transcripts, while during the night, the PER–CRY repressor complex is degraded, and CLOCK–BMAL1 can then activate a new cycle of transcription [43]. Our data suggest that in addition to skin, fibroblast and hair follicles [17,34,44], gene expression in oral mucosal tissue in humans display strong diurnal rhythms and therefore may be considered to serve as a biospecimen for developing circadian biomarkers. This is particularly important as unlike skin and fibroblast, collection of oral mucosal tissue allows for non-invasive multiple sampling.

These results provide a biological interpretation for clinical reports associating severity of OM with delivery time of radiotherapy [2]. In our own work using data from the electronic medical records of head and neck cancer patients, we found that the most severe cases of OM were associated with a radiation delivery time window of 11:30–15:00 [2], which overlaps with the peak time range of mRNA expression for several genes (***Table 2***) that we observed in healthy volunteers in the current study. During radiotherapy, OM is initiated by DNA damage and generation of reactive oxygen species, both of which trigger a series of interacting biological events including inflammation reactions, cell cycle deregulation, DNA damage response, and apoptosis [5]. Of all rhythmic genes identified, WEE1 and PER1 have been of most interest for further biomarker development due to their well-reported functions in these processes. WEE1 is a tyrosine kinase that phosphorylates CDC2 and prevents cell progression through G2/M and S cell cycle checkpoint into mitosis for DNA repair in response to DNA damage [45]. Preclinical and clinical studies have demonstrated encouraging antitumor effects of WEE1 inhibition [46]. Therefore, we expect expression of WEE1 would be negatively associated with radiation sensitivity and radiation-induced OM. The circadian pattern of WEE1 transcription has been reported in epithelium of the rat duodenum, ileum, jejunum, and colon [41], and is directly activated by the CLOCK–BMAL1 transcriptional complex in mouse liver [11]. On the other hand, ectopic expression of PER1 was found to inhibit WEE1 expression in human colon cancer cell lines [47]. Therefore CLOCK-BMAL1 and PER1 may regulate G2/M progression via WEE1. Circadian genes were also demonstrated to affect cell cycle progression at G1/S transition. PER1 inhibits p53-induced expression of p21, which prevents G1/S transition by inhibiting cyclin-dependent kinase activity including CDK4/6 [8,47]. These roles of PER1 on the G1/S and G2/M check points may free the cell cycle arrest from repairing DNA damage and lead to DNA damage induced apoptosis [45]. PER1 has also been directly involved in DNA damage response by interacting with the ATM and ATR DNA damage check point kinases or modulating activity of p53 [47]. In line with these roles in cell cycle progression and DNA damage response, the overexpression of PER1 has been shown to sensitize human cancer cells to ionizing radiation-induced apoptosis and cause significant growth reduction [47]. Therefore, we expect PER1 expression to be positively associated with radiation sensitivity and radiation-induced oral mucositis. In the current study, 8 (out of 11) participants had PER1 expression peaking 10:30~14:10, whereas expression of WEE1, a gene expected to be negatively associated with radio sensitivity, peaked during 13:30–19:00 in 8 participants; P53 did not show obvious diurnal pattern of expression. Although the exact mechanisms might be more complicated and need further study, this peak time range of PER1 expression (10:30–14:10) largely overlaps with the delivery time window of radiotherapy (11:30–15:00) associated with the most severe OM in head and neck cancer patients, which might partly interpret the underlying mechanism of the clinical observation [2].

Our results confirm the hypothesis that the peak times for cycling genes display inter-individual variability, which is one of the challenges for applying chronotherapy in the clinic. The range of peak times are gene specific: some cycling gene expression levels (CIART, PER1, PER3) had smaller range (about 8 hours) than others (about 11.5 hours for PER2 and 16 hours for ARNTL and WEE1). Our results suggested such variability may be partially contributed by individual characteristics: those with older ages tended to have later peak times for PER1 and PER3. This finding, however, is not in line with the documented phase shift toward a “morning” chronotype [48] and a phase advance in brain tissue [49] with aging; similar trend was observed in our data: earlier sleep time and earlier DLMO time associated with older age. But the age effect on the circadian clock timing in peripheral tissues may differ from the age effect on central clock or brain tissue. Further study is needed to verify or negate our finding on gene peak time and age. We also found that women tended to have later peak time of circadian gene expression than men (Supplementary Table 2), but the correlation with only one gene expression (PER3) reached borderline statistical significance. Given that we only included 3 men, these results need to be interpreted cautiously and need to be confirmed in a larger study. Further studies are also needed to understand more about the role of these genes associated with radio-sensitivity. These findings may help in developing an approach to guide individual timing of chronotherapy.

We did not find correlations between gene expression peak times and chronotype, sleep times or DLMO, except for negative correlation between DLMO and *PER1*. The lack of correlation with circadian or sleep timing suggest that these parameters representing central clock do not directly reflect the phases of gene expression in peripheral tissues. Overall, it emphasizes the importance of assessing the circadian timing of specific peripheral tissue that is subject to side effect of cancer therapy. However, given the large confidence intervals around the peak time estimates, potential confounding factors by age, as well as the small sample size (11 participants), it is possible that associations may exist and differ from what we observed. Waiting days (2–32 days) between actigraphy sleep measurement and sample collection days also limited the capability of finding correlation between sleep timing and gene peak timing. Studies with more frequent sample collection are needed to estimate the peak times of these genes more precisely and accurately. Better design is also needed to assess actigraphy sleep time that represent sample collection days.

Our study has several limitations. As mentioned above, the small sample size of this pilot study limited the statistical power for finding genes with diurnal rhythmic expression, and for assessing correlations between gene peak times and patient characteristics. Future studies with larger sample size are needed to confirm our findings. Our analysis was based on an assumption that mRNA expression follows a sinusoidal pattern with a 24-hour period. Violation of such assumption may also influence peak time estimation. To test this hypothesis, sample collections that cover more than 24-hour time interval will be ideal but collecting more than six consecutive samples from human oral mucosa is challenging. Our sampling design (every 4-hours for 6 samples) has been used in previous studies to determine diurnal rhythmicity [36]. Additionally, given that seven of the eight statistically significant genes belong to circadian regulation pathway and there is evidence of circadian expression of these genes in mammalian tissue, including gut epithelium [11,32,41,43], the assumption that mRNA expression of these genes follow a sinusoidal pattern in oral mucosa is likely to be true. There are other factors that can introduce random variations in measurement of RNA expression in genes, including differences in cell types present within the oral cavity (including bacterial), and sensitivity of the RNA expression assays. These factors can be minimized in the future by choosing more appropriate assay protocol. In this study, a standard whole transcriptome library preparation protocol was used. As a result, bacterial RNA from the microbiome may have accounted for a substantial proportion of the total sequenced RNA, which may have reduced the quantification accuracy of low expression genes. Alternative library preparation protocols, like capture-based RNA library preparation or message RNA specific library preparation with PolyA selection, may be more suitable. A low mapping rate to human genome ratio reflects a greater microbiome population or activity. Interestingly, the microbiome population and activity in our samples suggested a circadian pattern with a lower proportion of microbiome RNA during the day and higher at night (Supplementary Figure 1., Supplementary Figure 5.), which could be further investigated. Despite the study limitations, we were able to detect diurnal variations in a number of genes in the circadian pathway and cell cycle pathway. In addition, the correlations in peak times between the core circadian genes were consistent with their biological interactions and previous reports, strengthening the validity of our findings.

In conclusion, we found diurnal variations in mRNA expression patterns for genes involved in circadian rhythm regulation and cell cycle progression, including a novel finding of *WEE1* cycling in human oral mucosa. Identified genes may serve as candidates for development of time-varying biomarkers using oral mucosal tissue collected by non-invasive brush samples. Such biomarkers, combined with other patient characteristics (such as age, sex), merit further study, particularly in cancer patients, to develop an approach for estimating individual treatment time windows for radiotherapy, a critical challenge in the application of cancer chronotherapy. Finally, although this study was motivated by reducing radiation-induced OM, results have broader implication for cancer chronotherapy in general.

## Additional Files

The additional files for this article can be found as follows:

10.5334/jcr.213.s1 Supplementary Figure 1. Boxplot for RIN score by sample collection time order. Samples C01-C06 were collected at approximately 10:00, 14:00, 18:00, 22:00, 2:00 and 6:00, respectively.

10.5334/jcr.213.s2 Supplementary Figure 2. **(a)** Gene-feature composition plot describing genomic content distribution of oral mucosal samples by each participant and by sample collection time order. Samples C01-C06 were collected at approximately 10:00, 14:00, 18:00, 22:00, 2:00 and 6:00, respectively. Higher CDS exon content is expected from good quality RNAseq samples. **(b)** Percentage of mapped gene region by participant and sample collection time order. Samples C01–C06 were collected at approximately 10:00, 14:00, 18:00, 22:00, 2:00 and 6:00, respectively.

10.5334/jcr.213.s3 Supplementary Figure 3. Gene body coverage of housekeeping genes for each collected sample by individual. Samples C01-C06 were collected at approximately 10:00, 14:00, 18:00, 22:00, 2:00 and 6:00, respectively.

10.5334/jcr.213.s4 Supplementary Figure 4. Scatter plots showing correlation of age with gene peak times, sleep times and DLMO time.

10.5334/jcr.213.s5 Supplementary Figure 5. Boxplot for mapping read percentage by sample collection time order. Samples C01-C06 were collected at approximately 10:00, 14:00, 18:00, 22:00, 2:00 and 6:00, respectively.

10.5334/jcr.213.s6 Supplementary Table 1. Results of all candidate genes in the three pathways and the 108 genes genome-wide with p-value < 0.05.

10.5334/jcr.213.s7 Supplementary Table 2. Peak time of gene mRNA expression by sex.
