# Supplementary material for: Genes Relevant to Tissue Response to Cancer Therapy Display Diurnal Variation in mRNA Expression in Human Oral Mucosa
Source: J Circadian Rhythms. 2021 Jun 17;19:8. doi: 10.5334/jcr.213 (PMC8231453; doi:10.5334/jcr.213)

Supplementary Figure 2a. Gene-feature composition plot describing genomic content distribution of oral mucosal samples by each participant and by sample collection time order. Samples C01-C06 were collected at approximately 10:00, 14:00, 18:00, 22:00, 2:00 and 6:00, respectively. Higher CDS exon content is expected from good quality RNAseq samples.

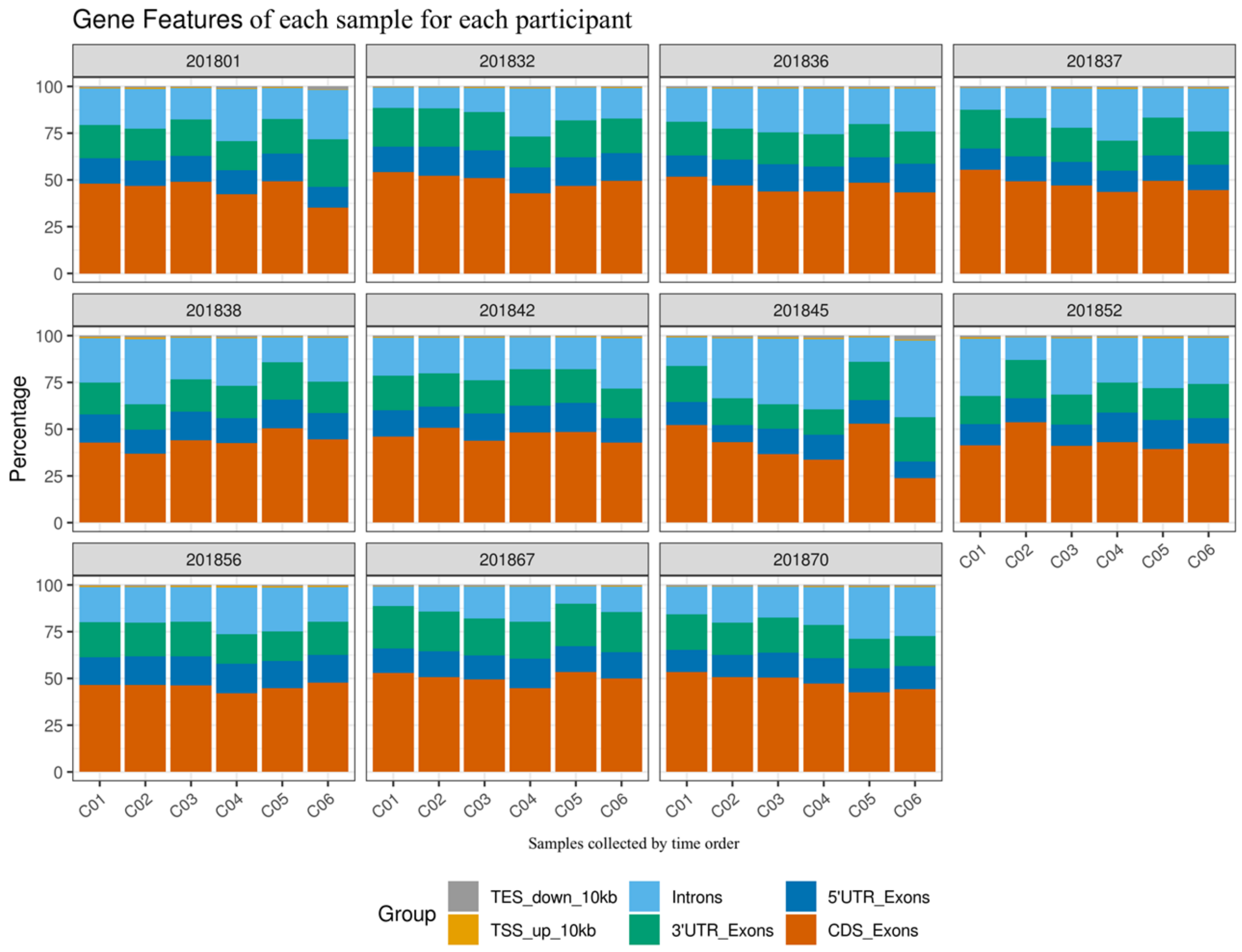

Supplementary Figure 2b. Percentage of mapped gene region by participant and sample collection time order. Samples C01-C06 were collected at approximately 10:00, 14:00, 18:00, 22:00, 2:00 and 6:00, respectively.

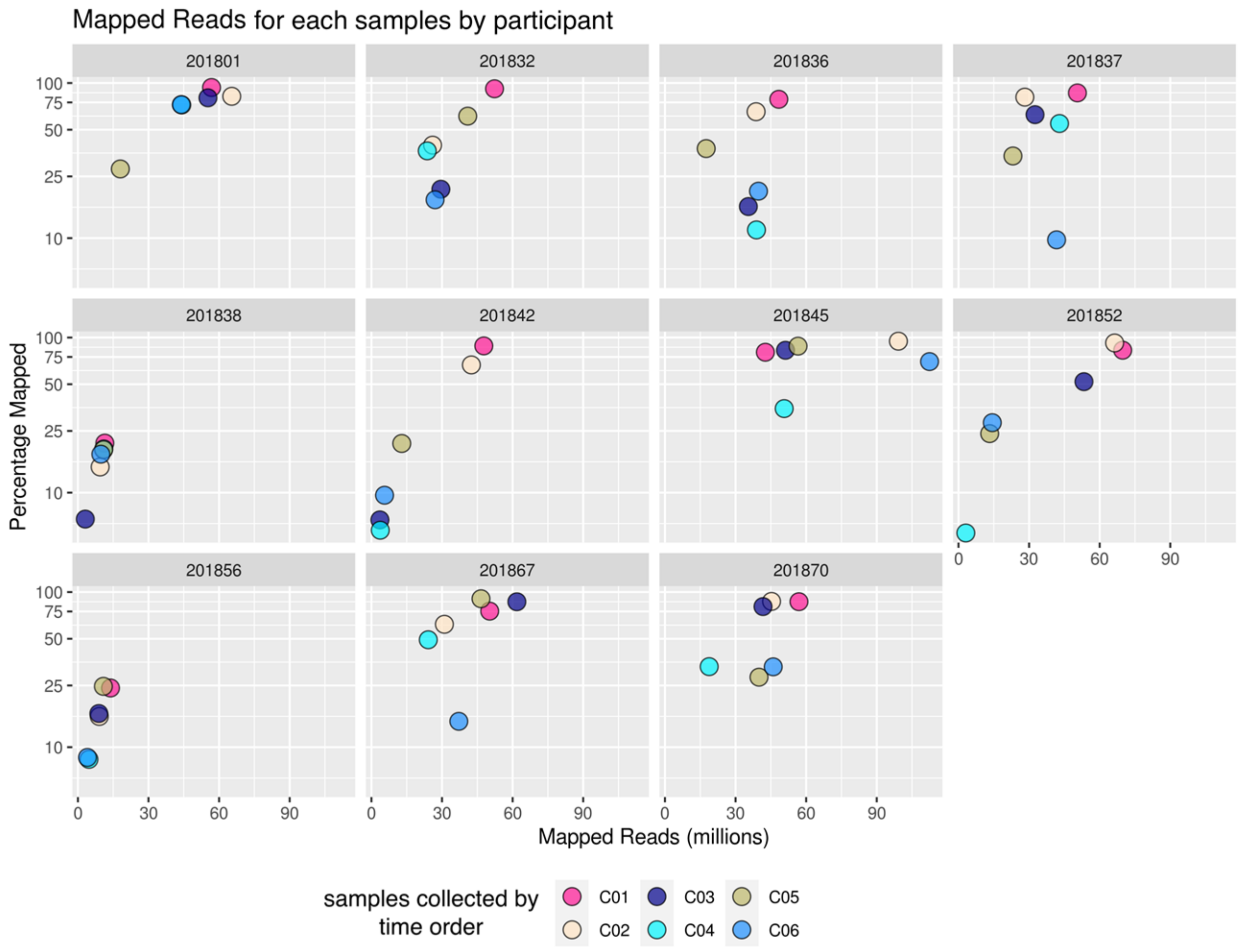

Supplement: Supplementary Figure 2. — (a) Gene-feature composition plot describing genomic content distribution of oral mucosal samples by each participant and by sample collection time order. Samples C01-C06 were collected at approximately 10:00, 14:00, 18:00, 22:00, 2:00 and 6:00, respectively. Higher CDS exon content is expected from good quality RNAseq samples. (b) Percentage of mapped gene region by participant and sample collection time order. Samples C01–C06 were collected at approximately 10:00, 14:00, 18:00, 22:00, 2:00 and 6:00, respectively. [file jcr-19-213-s2.pdf]
