# Supplementary material for: Genes Relevant to Tissue Response to Cancer Therapy Display Diurnal Variation in mRNA Expression in Human Oral Mucosa
Source: J Circadian Rhythms. 2021 Jun 17;19:8. doi: 10.5334/jcr.213 (PMC8231453; doi:10.5334/jcr.213)

Supplementary Figure 4. Scatter plots showing correlation of age with gene peak times, sleep times and DLMO time.

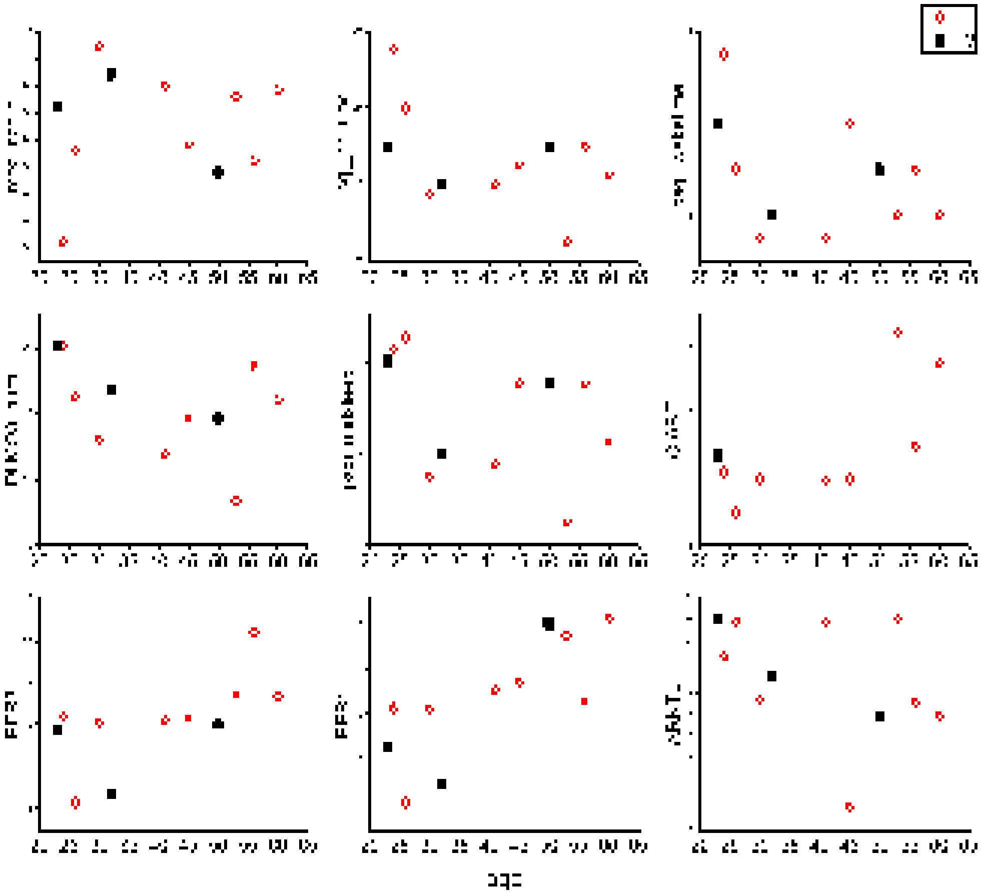

Supplement: Supplementary Figure 4. — Scatter plots showing correlation of age with gene peak times, sleep times and DLMO time. [file jcr-19-213-s4.pdf]
