# Supplementary material for: Genes Relevant to Tissue Response to Cancer Therapy Display Diurnal Variation in mRNA Expression in Human Oral Mucosa
Source: J Circadian Rhythms. 2021 Jun 17;19:8. doi: 10.5334/jcr.213 (PMC8231453; doi:10.5334/jcr.213)

Supplementary Figure 5. Boxplot for mapping read percentage by sample collection time order. Samples C01-C06 were collected at approximately 10:00, 14:00, 18:00, 22:00, 2:00 and 6:00, respectively.

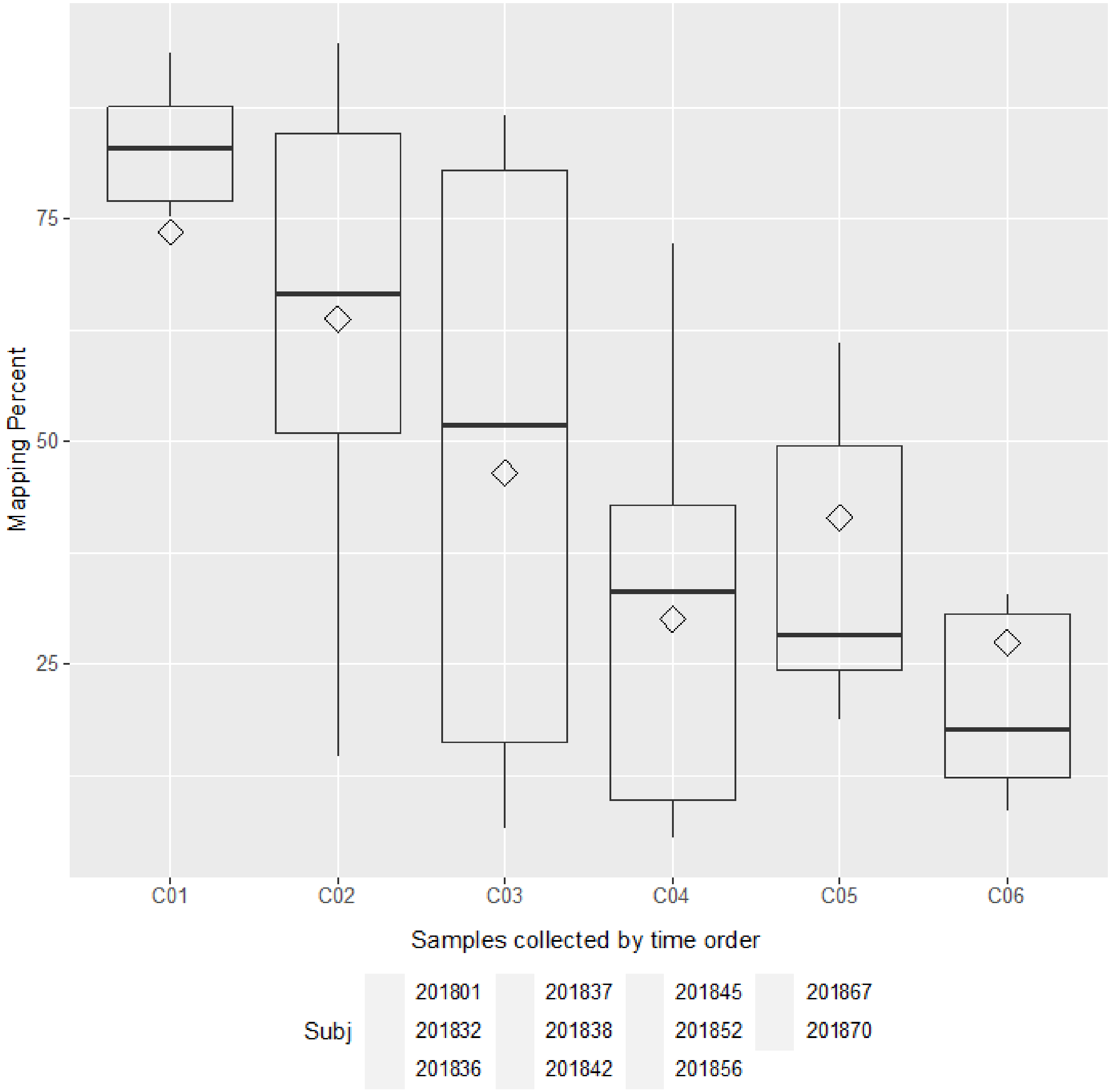

Supplement: Supplementary Figure 5. — Boxplot for mapping read percentage by sample collection time order. Samples C01-C06 were collected at approximately 10:00, 14:00, 18:00, 22:00, 2:00 and 6:00, respectively. [file jcr-19-213-s5.pdf]
