# Supplementary material for: Genes Relevant to Tissue Response to Cancer Therapy Display Diurnal Variation in mRNA Expression in Human Oral Mucosa
Source: J Circadian Rhythms. 2021 Jun 17;19:8. doi: 10.5334/jcr.213 (PMC8231453; doi:10.5334/jcr.213)
Supplement: Supplementary Table 2. — Peak time of gene mRNA expression by sex. [file jcr-19-213-s7.pdf]

Supplementary Table 2. Peak time of gene mRNA expression by sex

Genes	Women (n = 8)	Men (n = 3)
CIART	11.0 (1.9)	8.2 (2.2)
PER1	10.9 (2.4)	9.8 (3.6)
PER2	13.3 (2.3)	10.4 (4.2)
PER3[*]	10.6 (2.2)	8.8 (1.8)
WEE1	12.8 (4.8)	13.4 (6.1)
ARNTL	19.9 (5.3)	17.8 (4.2)

[*]P value = 0.06 for *PER3*, and >0.1 for other genes.
